# Synergistic Role of Temperature and Salinity in Aggregation of Nonionic Surfactant-Coated Silica Nanoparticles

**DOI:** 10.1021/acs.langmuir.3c00432

**Published:** 2023-04-13

**Authors:** Yingzhen Ma, Christian Heil, Gergely Nagy, William T. Heller, Yaxin An, Arthi Jayaraman, Bhuvnesh Bharti

**Affiliations:** † Cain Department of Chemical Engineering, Louisiana State University, Baton Rouge, Louisiana 70803, United States; ‡ Department of Chemical and Biomolecular Engineering, University of Delaware, Newark, Delaware 19716, United States; § Neutron Scattering Division, Oak Ridge National Laboratory, Oak Ridge, Tennessee 37831, United States

## Abstract

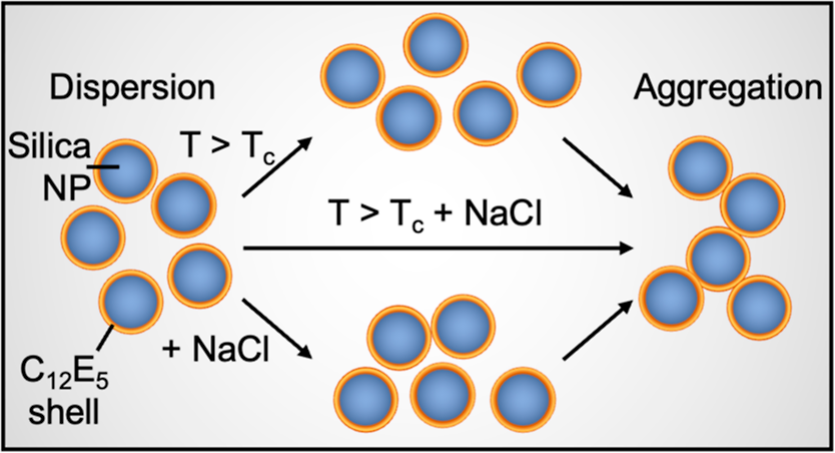

The adsorption of nonionic surfactants onto hydrophilic nanoparticles (NPs) is anticipated to increase their stability in aqueous medium. While nonionic surfactants show salinity- and temperature-dependent bulk phase behavior in water, the effects of these two solvent parameters on surfactant adsorption and self-assembly onto NPs are poorly understood. In this study, we combine adsorption isotherms, dispersion transmittance, and small-angle neutron scattering (SANS) to investigate the effects of salinity and temperature on the adsorption of pentaethylene glycol monododecyl ether (C_12_E_5_) surfactant on silica NPs. We find an increase in the amount of surfactant adsorbed onto the NPs with increasing temperature and salinity. Based on SANS measurements and corresponding analysis using computational reverse-engineering analysis of scattering experiments (CREASE), we show that the increase in salinity and temperature results in the aggregation of silica NPs. We further demonstrate the non-monotonic changes in viscosity for the C_12_E_5_–silica NP mixture with increasing temperature and salinity and correlate the observations to the aggregated state of NPs. The study provides a fundamental understanding of the configuration and phase transition of the surfactant-coated NPs and presents a strategy to manipulate the viscosity of such dispersion using temperature as a stimulus.

## 1 Introduction

The stability of nanoparticles (NPs) in aqueous solvents is one of the most important criteria considered while formulating dispersions for applications in biomedicine, water treatment, and personal care products.^1−7^ Over the past century, adsorption of surfactants on NPs has emerged as a generic approach for imparting long-term stability to the NPs.^8,9^ Surfactants can adsorb onto NPs through a variety of interactions including hydrogen bonding, hydrophobic interactions, and electrostatic attraction between the surfactant molecules and the NPs.^10−12^ The choice of the surfactant for stabilizing NPs is generally dependent on the surface chemistry of the NPs, the solvent characteristics, and other application-specific requirements.^8,9,13,14^ Nonionic surfactants are one sub-group of surfactants widely used for stabilizing NPs in aqueous solvents. These surfactants have a neutral hydrophilic headgroup and a hydrophobic tail. The nonionic surfactants readily adsorb onto hydrophilic NPs to induce a steric barrier, thus impeding NP aggregation. While significant literature exists on the use of nonionic surfactants for stabilizing hydrophilic NPs,^15−18^ the relationship between the NP stability and aqueous solvent conditions such as temperature and salinity is poorly understood. This knowledge gap exists due to the non-trivial dependence of the surfactant phase behavior on temperature and salinity, thus obscuring a clear link between NP stability and solvent conditions. This article aims to provide a better understanding of the relationship of solvent temperature and salinity with the stability of nonionic surfactant-coated hydrophilic NPs.

*n*-Alkyl poly(oxyethylene) ethers, represented as C_*n*_E_*m*_, are a class of nonionic surfactants consisting of a hydrocarbon tail (C_*n*_) and an ethoxylated headgroup (E_*m*_).^18^ These surfactants are widely used for the synthesis and stabilization of NPs in aqueous and nonaqueous solvents.^19−21^ In water, these surfactants show a temperature-dependent phase behavior, where these molecules phase-separate above a characteristic temperature known as the cloud point (*T*_c_).^18,22^ The cloud-point transition occurs due to the dehydration of the surfactant headgroup above *T*_c_, resulting in a decrease in the surfactant’s solubility in the aqueous phase and establishing a liquid–liquid equilibrium between a surfactant-rich and a surfactant-depleted phase.^23^ The *T*_c_ is strongly dependent on the number of carbon atoms in the tail and the ethoxy groups within the headgroup (i.e., *n* and *m* in C_*n*_E_*m*_) and other additives including electrolytes.^24^

The C_*n*_E_*m*_ surfactants adsorb onto hydrophilic solid–liquid interfaces via anchoring of their headgroup and forming a bilayer on the surface.^25^ Surprisingly, an increase in dispersion temperature above *T*_c_ leads to an increase in the amount of surfactant adsorbed on the hydrophilic surfaces.^26−28^ This anomalous temperature dependence of the adsorption behavior of C_*n*_E_*m*_ is the result of the breaking of the hydrogen bonds between the surfactant and water (solvent), leading to an increase in the rotational entropy of the surfactant molecules.^27^ Such a change in thermodynamic characteristics manifests itself in the form of increasing adsorption of free surfactant molecules from bulk onto the assemblies pre-adsorbed on the hydrophilic surface.^28^ Previously, we have shown that such anomalous adsorption behavior of the C_*n*_E_*m*_ surfactants enables a dynamic partitioning of the surfactant molecules between the spatially confined pore space and the bulk aqueous solvent.^28^ However, the impact of such anomalous temperature dependence of adsorption of the C_*n*_E_*m*_ surfactants on the stability of hydrophilic NPs remains unknown and is the focus of this article. Here, we use pentaethylene glycol monododecyl ether (C_12_E_5_) as a model surfactant to investigate the effects of temperature and salinity on its ability to adsorb onto silica NPs and evaluate the resulting change in NP dispersion stability. While temperature impacts the hydration state of the nonionic surfactant headgroup, dispersion salinity primarily alters the interactions among the hydrocarbon surfactant tails,^29,30^ resulting in a complex NP phase behavior dependence on the two parameters.

Silica NPs have wide applicability in industry, ranging from additives in food to surface coatings.^31−36^ Owing to their well-known surface chemistry,^37,38^ silica NPs are an excellent model system to investigate fundamental processes such as molecular adsorption and NP stabilization. Here, we use silica NPs as a model to investigate the impact of solvent temperature and salinity on the adsorption of C_12_E_5_ and the corresponding colloidal stability. The mechanism of adsorption of C_*n*_E_*m*_ surfactants onto silica surfaces (Si–OH and Si–O^–^) is well-established,^1,16,39^ where the H-bonding between the ethoxylated headgroup and the silica surface drives the adsorption process. Recently, nonionic surfactants have been shown to induce aggregation of silica NPs in aqueous dispersions.^40^ The aggregation process was driven by the depletion attraction between the NPs due to the presence of excess, non-adsorbed surfactants in the dispersion. However, the effect of solvent conditions on the stability of C_*n*_E_*m*_-coated silica NPs in the absence of any excess surfactants (i.e., depletion attraction) is unknown.

In this article, we investigate the effects of increasing solvent temperature and salinity on the adsorption behavior of the C_12_E_5_ surfactant onto silica NPs and quantify the corresponding change in the stability of the NPs. We use adsorption isotherms, dispersion transmittance, and small-angle neutron scattering (SANS) as experimental tools to determine the temperature- and salinity-dependent self-assembled state of C_12_E_5_ on the silica and the corresponding effects on the NP stability. We identify a clear synergy between these two solvent parameters and show that the simultaneous increase in temperature and salinity enhances the surfactant adsorption and silica aggregation processes. Furthermore, we quantify the changes in the viscosity of the silica–C_12_E_5_ mixture upon increasing the salinity and temperature. Uncovering the interactions governing the stability and phase behavior of NP–surfactant complexes is fundamental to designing nanomaterials with tunable interfacial and bulk properties, such as transparency and viscosity.^41,42^ This study provides a better understanding of the interactions governing the dispersed state of nonionic surfactant-coated NPs in aqueous solvents and provides a link between dynamically tunable solvent conditions and NP stability.

## 2 Materials and Methods

### 2.1 Materials

Following is the list of chemicals and materials used in the study along with the supplier and purity: Ludox-TMA colloidal silica (Sigma-Aldrich, wt % ∼30%), C_12_E_5_ (Sigma-Aldrich, purity ≥98%), D_2_O (Sigma-Aldrich, 99.9% D), NaCl (Sigma-Aldrich, purity ≥99%), and cellulose dialysis membrane (Spectrum Spectra/Por, molecular weight cutoff 12–14 kDa). All experiments were performed using 18.1 MΩ water from the Elga Flex 3 system.

### 2.2 Adsorption Isotherms

We used the solvent depletion method to experimentally quantify the amount of C_12_E_5_ surfactant adsorbed on the silica NPs and obtained adsorption isotherms at 20, 30, and 40 °C.^43^ We investigated the effect of increasing temperature and salinity on the adsorption of C_12_E_5_ to silica NPs using sodium chloride (NaCl) as a model 1:1 electrolyte. In a typical adsorption experiment, increasing concentrations of C_12_E_5_ were added to dispersions containing 1 wt % silica NPs. NaCl (powder) was added to these dispersions to obtain the desired molarity. The silica–C_12_E_5_ mixture was equilibrated at the target temperature (*T*) for 24 h in an orbital shaker. The silica NPs with adsorbed C_12_E_5_ were separated from the dispersion using centrifugation at 18,000*g* for 2 h, where *g* is the gravitational acceleration. The concentration of the unadsorbed C_12_E_5_ in the supernatant was determined using surface tension calibration curves at the corresponding NaCl concentration, as shown in Figure S1. The surface tension was measured using optical tensiometry (Theta from Biolin Scientific), and the droplet shape was analyzed by the Young–Laplace equation.^28,44^ Note that the presence of any residual silica NPs in the supernatant does not significantly impact surface tension, as the silica NPs used in the study are highly hydrophilic and remain in the aqueous phase. All calibration curves were measured at 20 °C. For determining the unadsorbed concentration of C_12_E_5_ for isotherms at *T* > 20 °C, the supernatant was first cooled, and then, the surface tension was measured at 20 °C. The amount of C_12_E_5_ adsorbed on silica NPs (Γ) was calculated as
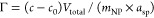
where *c* is the initial concentration of the surfactant in the silica–C_12_E_5_ mixture, *c*_0_ is the equilibrium concentration of the surfactant in the bulk solvent, *V*_total_ is the total volume of aqueous solution, and *m*_NP_ and *a*_sp_ are the mass and specific surface area of the silica NPs, respectively.

### 2.3 Transmittance

Transmittance of the aqueous silica–C_12_E_5_ mixtures were determined on the Litesizer 500 (Anton Paar) using disposable cuvettes with a path length of 5 mm. The cuvettes were completely sealed to prevent contamination and evaporation of the solvent during the measurement. In a typical experimental run, the temperature was increased from 20 to 60 °C with 5 °C intervals. The sample was equilibrated for 2 min before measuring the transmittance at a given *T* using a 40 mW laser of wavelength 658 nm.

### 2.4 Small-Angle Neutron Scattering

The SANS experiments were conducted on an EQ-SANS instrument at SNS of the Oak Ridge National Laboratory (ORNL).^45^ The SANS measurements were performed in quartz cuvettes of path length 1 mm using a temperature-controlled sample holder. We used three instrument configurations for the SANS measurements, namely, sample-to-detector distance/wavelength: 8 m/10 Å, 4 m/6 Å, and 2.5 m/2.5 Å, which provided access to the scattering vector,  in the range 0.08 < *q* < 3 nm^–1^, where λ is the wavelength of the collimated neutrons and θ is the scattering angle. Data were reduced according to the standard procedures that were implemented in *drtsans* software.^46^ The two-dimensional (2D) scattering patterns were radially averaged to obtain 1D scattering intensity *I*(*q*) profiles. Discussion on the solvent contrast scenarios used during the SANS measurements is provided later in the article, and further details on the SANS experiments can be obtained from our previous publications.^28,47,48^

## 3 Results and Discussion

### 3.1 Characterization of Silica NPs

**Figure 1:**
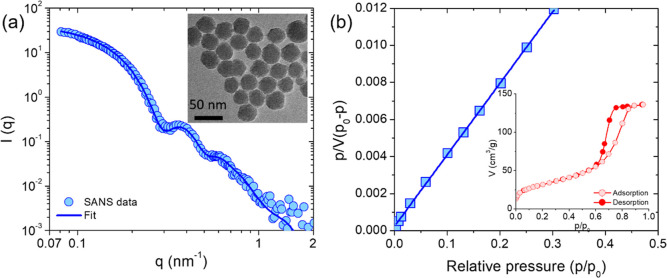
(a) SANS profile of silica NPs in D_2_O at 20 °C (circles). Line is the best fit to the experimental data using the form factor of the sphere with log-normal size distribution. Inset: TEM image showing the spherical shape of the silica NPs of diameter 30 nm. (b) BET plot of the N_2_ gas adsorption isotherm for silica NPs. Inset: adsorption–desorption isotherm for the N_2_ gas on silica NPs.

We used commercially available Ludox-TMA colloidal silica as model hydrophilic NPs. The silica dispersion was dialyzed for 7 days in deionized (DI) water to remove any undesired foreign molecules in the dispersion. The dialysis was performed using a cellulose membrane of molecular weight cutoff of 12–14 kDa, and the DI water was changed every 24 h. The size and polydispersity of silica NPs were determined using SANS and transmission electron microscopy (TEM, JOEL JEM 2011). For SANS, the solvent of the silica NP dispersion was changed to D_2_O (to minimize incoherent scattering) by performing the dialysis in D_2_O instead of H_2_O. The SANS profile for 1 wt % silica NPs in D_2_O is shown in Figure 1a. The experimental scattering profile is fitted using the form factor of spheres with log-normal particle size distribution. The diameter (σ) and polydispersity index for silica NPs obtained from SANS data fitting were 30 nm and 0.1, respectively, which agree with TEM imaging (Figure 1a inset). The specific surface area of silica NPs (*a*_sp_) was determined by N_2_ gas adsorption, as shown in Figure 1b (Micromeritics, ASAP 2020). The N_2_ adsorption isotherm was analyzed in the relative pressure regime of 0.0 < *p*/*p*_0_ < 0.3 using the Brunauer–Emmett–Teller (BET) model, and the *a*_sp_ for silica NPs was estimated to be 115 m^2^ g^–1^.

### 3.2 Adsorption of C_12_E_5_ on Silica NPs

The *T*_c_ of C_12_E_5_ in water is strongly dependent on the dispersion salinity. We find that for 2.6 mM C_12_E_5_ dissolved in water, the value of *T*_c_ decreases from 38 °C at 0 mM NaCl to 32 °C at 2 mM and 5 mM NaCl (Figure S2). The decrease in *T*_c_ highlights the inherent changes occurring in the surfactant molecules upon increasing temperature and the addition of NaCl. Here, we further investigate the effect of increasing temperature and dispersion salinity on the amount of C_12_E_5_ surfactant bound to the surface of silica NPs using adsorption isotherms. The isotherms for C_12_E_5_ adsorption on silica NPs were measured using the solvent depletion method (Section 2) in presence of 0, 2, and 5 mM NaCl at *T* = 20, 30, and 40 °C.

**Figure 2:**
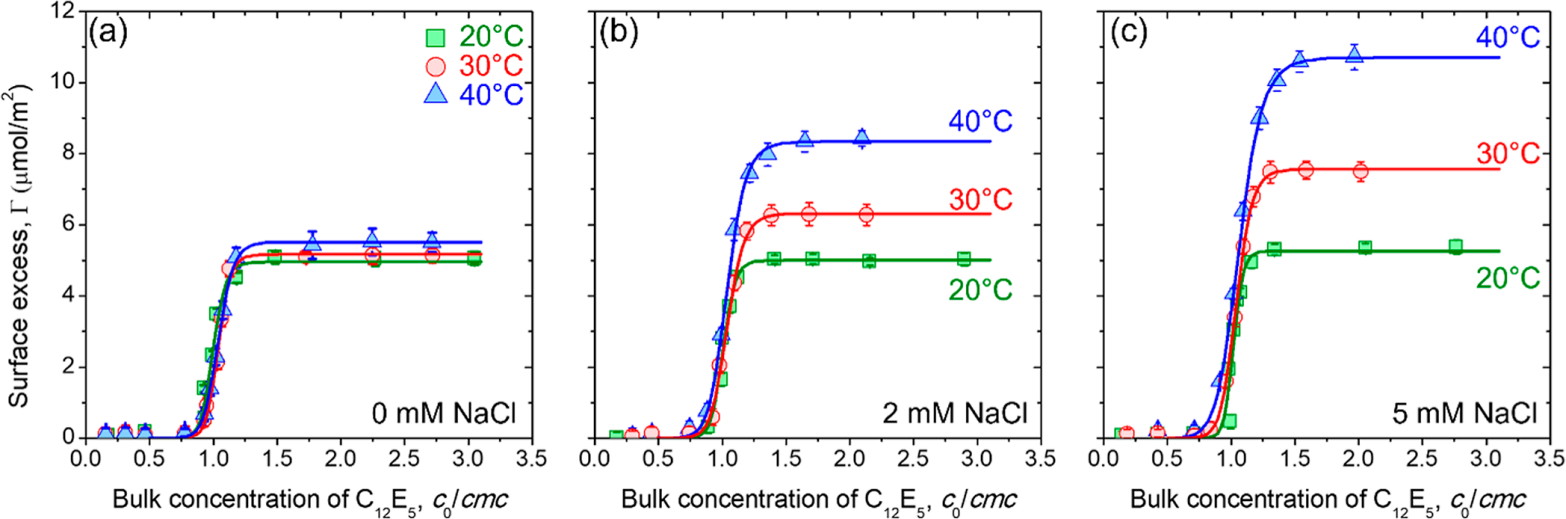
Isotherms for the adsorption of C_12_E_5_ on silica NPs containing (a) 0, (b) 2, and (c) 5 mM NaCl at 20, 30, and 40 °C. The points are the experimentally measured values, and solid lines represent the best fit using the Gu–Zhu adsorption model given by eq 1. The maximum surface excess of the surfactant bound to the silica surface increases upon simultaneously increasing dispersion salinity and temperature.

The adsorption of C_12_E_5_ onto silica NPs remains unaffected by either increasing the temperature or dispersion salinity independently. However, a simultaneous increase in the temperature and dispersion salinity drives an increase in the amount of the surfactant adsorbed onto silica NPs, highlighting the synergistic role of the two parameters in enhancing surfactant adsorption. At all tested temperatures and salinities, the adsorption isotherms show a sigmoidal shape, which is characteristic of the cooperative adsorption behavior.^39^ Such a cooperative adsorption of C_12_E_5_ on silica NPs is well-known, where a characteristic minimum number of surface-bound surfactant molecules enable a rapid increase in surface adsorption.^1,28^ The amount of the adsorbed C_12_E_5_ onto silica NPs reaches a constant maximum value (Γ_*m*_), which is a signature of the fixed number of binding sites available on the silica surface. Here, we use the Gu–Zhu model to represent the observed cooperative adsorption as^49^
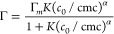
1where Γ is the amount of surfactant adsorbed, cmc is the critical micelle concentration, *K* is the equilibrium adsorption constant which is proportional to the binding energy of the surfactant to the surface, and α is the cooperativity factor. The experimentally measured adsorption isotherms at different temperatures and salinities are fitted using the Gu–Zhu model. The best fit curves are shown in Figure 2, and the fit parameters are given in Table 1.

**Table 1:** Fit Parameters for the C_12_E_5_ Adsorption Onto Silica NPs^a^

Conc. of NaCl (mM)	*T* (°C)	Γ_m_ (μmol m^–2^)	α	*K* (mM^–1^)
0	20	5.0	26	0.51
	30	5.1	25	0.51
	40	5.4	23	0.50
2	20	5.1	24	0.51
	30	6.2	19	0.50
	40	8.4	16	0.50
5	20	5.2	23	0.53
	30	7.6	17	0.50
	40	10.7	12	0.49

a The parameters are obtained by fitting the experimental data using the Gu-Zhu model.

The change in the hydration state of the surfactant headgroup upon increasing temperature and the increase in tail–tail hydrophobic interaction upon increasing salinity drive the observed changes in adsorption behavior of C_12_E_5_ onto silica NPs.^26−30^ The rapid increase in Γ with *c*_0_/cmc remains independent of the dispersion temperature and salinity (Figure 2a–c). This invariance is also reflected in the fitting results of the experimental data using the Gu-Zhu model, which shows a near-constant value for adsorption constant *K* = 0.5. This observation contrasts with the previous reports (including ours) on the adsorption of C_6_E_3_ and C_8_E_4_ onto silica surfaces,^26,28^ where an increase in the value of *K* with temperature was observed. In previous studies with C_6_E_3_ and C_8_E_4_, the binding energy increases with increasing temperature due to the loss of water in the surfactant headgroup, promoting the adsorption of molecules onto the silica surface. However, in the present case of C_12_E_5_, the adsorption free energy at room temperature is anticipated to be significantly larger than that of C_6_E_3_ and C_8_E_4_ due to the larger number of ethoxy groups in the surfactant headgroup. Therefore, the surfactant molecules are relatively strongly bound to the surface, and the temperature (in the tested range) does not seem to significantly impact the binding energy of surfactants directly adsorbed on the surface of silica. Note that C_12_E_5_ forms a bilayer on the surface of silica NPs,^1^ and the lack of a significant change in the value of *K* highlights that the adsorption free energy of surfactant molecules directly bound to the silica surface remains nearly unaltered upon increasing dispersion salinity and temperature (in the tested range).

At 20 °C, the adsorption of C_12_E_5_ molecules on the silica surface results from the hydrogen bonding between the surfactant headgroup and silica NPs.^15,17^ Interestingly, we find that the increasing temperature alone does not increase Γ_m_ (Figure 2a and Table 1). However, a significant increase in the maximum surface excess of C_12_E_5_ can be observed upon increasing the temperature of the dispersions containing NaCl. We find that the cooperativity factor α also remains nearly constant for either increasing temperature or salinity, but it decreases upon simultaneously increasing temperature and dispersion salinity. The following two conclusions can be made based on the observed increase in Γ_*m*_ and decrease in α: (1) Larger amounts of surfactant can be loaded on the NPs with increasing temperature and salinity and (2) an increase in the amount of the adsorbed surfactant is the result of increasing surfactant–surfactant attraction. It is well-established that the addition of the electrolyte promotes hydrophobic interactions among molecules dispersed in aqueous solvents.^50,51^ In our case, an increase in surface adsorption with temperature at near constant *K* and a decrease in α suggests that the dehydration of the surfactant molecules leads to a closer packing of the C_12_E_5_ on the silica surface. This increase could be due to ‘“condensation” of the surfactant molecules within the mesopores formed upon the aggregation of the NPs (discussed later). The performed adsorption isotherms and SANS measurements do not allow for resolving the sites for the surfactant upon increasing temperature. Future work using calorimetry and molecular dynamic simulations will clarify the mechanism of the increase in adsorption. In addition to the change in the amount of surfactant adsorbed onto silica, adjusting solution temperature and dispersion salinity had a profound impact on the stability of the NPs.

### 3.3 Aggregation of C_12_E_5_-Coated Silica NPs

**Figure 3:**
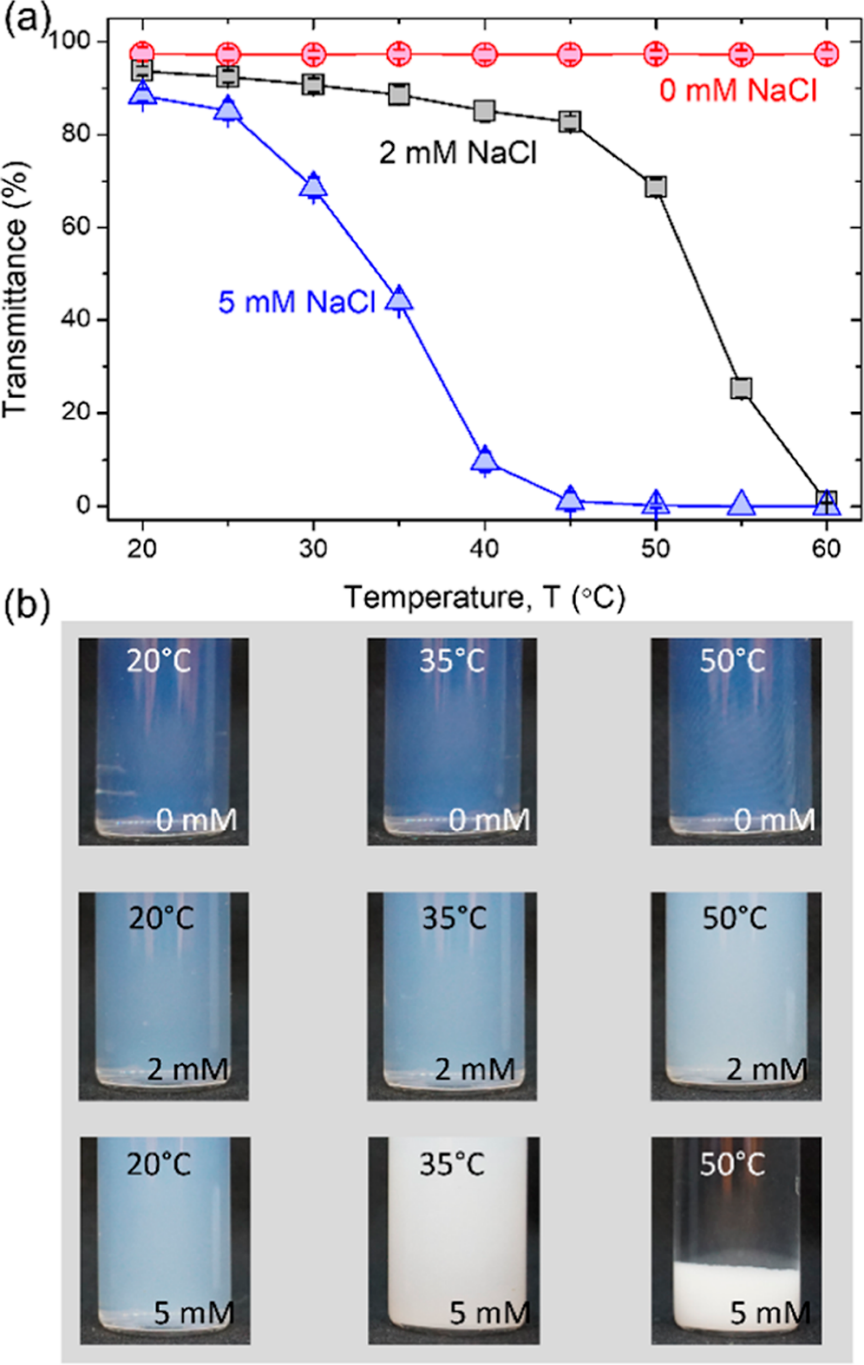
(a) Transmittance of C_12_E_5_-coated silica NPs with 0, 2, and 5 mM NaCl upon increasing temperatures from 20 to 60 °C. (b) Photographs of the dispersions highlighting the change in transmittance for the aqueous dispersion upon increasing salinity and temperature.

Adsorption of the surfactant on NPs is generally associated with improved NP stability in aqueous solvents. However, we find that the simultaneous increase in temperature and salinity promotes irreversible aggregation of C_12_E_5_-coated silica NPs. Here, we perform transmittance experiments to investigate the change in the dispersed state of silica–C_12_E_5_ mixtures as a function of temperature with the addition of NaCl. In a typical experiment, 1 wt % silica NP dispersion was mixed with a constant amount of C_12_E_5_ surfactant in the presence of 0, 2, and 5 mM NaCl. The amount of C_12_E_5_ used in the experiments was equivalent to 90% of the maximum surface excess, that is, Γ = 0.9Γ_m_ = 4.5 μmol m^–2^ of silica NPs in deionized water at 20 °C. Thus, the added C_12_E_5_ molecules existed primarily in the adsorbed state on silica NPs, and only a small fraction of added surfactant was present in the bulk solvent. The transmittance of the silica–C_12_E_5_ mixture in the presence of salt at different temperatures is shown in Figure 3.

At 0 mM NaCl, the transmittance shows a temperature-independent behavior. It is identical to that of pure silica NP dispersion (Figure S3) in the absence of the surfactant, highlighting the stability of both the surfactant-coated and uncoated silica NPs in low-salinity dispersions. Upon increasing the NaCl concentration to 2 mM, the transmittance of the surfactant-coated silica NPs shows a rapid decrease from ∼70% at 50 °C to ∼0% at 60 °C. This decrease in transmittance highlights the aggregation of C_12_E_5_-coated silica NPs. At *T* > 50 °C and 2 mM NaCl, the strong hydrophobic interactions from the dehydrated headgroup of C_12_E_5_ and their tails drive the aggregation of silica NPs. At 5 mM NaCl, the transmittance decreases rapidly with increasing temperature, and transmittance at 5 mM is lower than the transmission of C_12_E_5_-coated silica NP dispersion at 2 mM at equivalent temperature (Figure 3a,b). Upon increasing the NaCl concentration further to 5 mM, C_12_E_5_ becomes more dehydrated and causes stronger hydrophobic attraction between silica NPs. Additional screening of the electrical double-layer repulsion between the core particle could also contribute to the observed aggregation of the silica NP, but the screening is not the only reason for the aggregation of silica NPs. This assertion can be confirmed by determining the change in transmittance of a bare silica NP dispersion across the temperature range in 5 mM NaCl. The transmittance remains nearly constant in the tested temperature range (Figure S3). Thus, the surface-adsorbed C_12_E_5_ surfactant plays a critical role in driving the aggregation behavior of silica NPs. In summary, the transmittance experiments show that the temperature and salinity provide a synergistic effect on the dehydration of C_12_E_5_ adsorbed on the silica surface. The dehydrated state of the adsorbed surfactant and corresponding strength of the hydrophobic attractions between C_12_E_5_-coated silica NPs are amplified with high electrolyte concentration.

### 3.4 Structural Characterization of Silica–C_12_E_5_ Using SANS

The adsorption isotherms provided direct information on the change in the amount of C_12_E_5_ adsorbed onto silica NPs, and the transmittance measurements allowed for asserting the presence/absence of NP aggregates. However, no information could be obtained on the effect of temperature and salinity on the structure of C_12_E_5_ on the silica NPs and corresponding NP aggregates. Here, we perform SANS experiments to uncover the changes occurring in the C_12_E_5_-coated silica NP dispersions upon altering the temperature and dispersion salinity. The SANS experiments were performed in a H_2_O/D_2_O solvent mixture, providing the following two solvent contrast scenarios.

#### 3.4.1 Core Contrast-Matched

For the solvent H_2_O/D_2_O mixture matching the scattering length density (SLD) of core silica NPs, the neutrons scatter solely from the surfactant molecules adsorbed onto silica NPs. Therefore, the SANS profiles provide direct and exclusive information on the changes occurring in the surfactant adsorbed onto silica NPs upon altering temperature and dispersion salinity.

#### 3.4.2 Shell Contrast-Matched

The second contrast scenario used in the study is where the solvent H_2_O/D_2_O mixture matches the SLD of the surfactant shell. Such contrast matching enables the quantification of the changes in the silica NP structural arrangement driven by the changes in the temperature and dispersion salinity.

### 3.5 Structure of the Surfactant Shell on Silica NPs: SANS with Core Contrast-Matched

**Figure 4:**
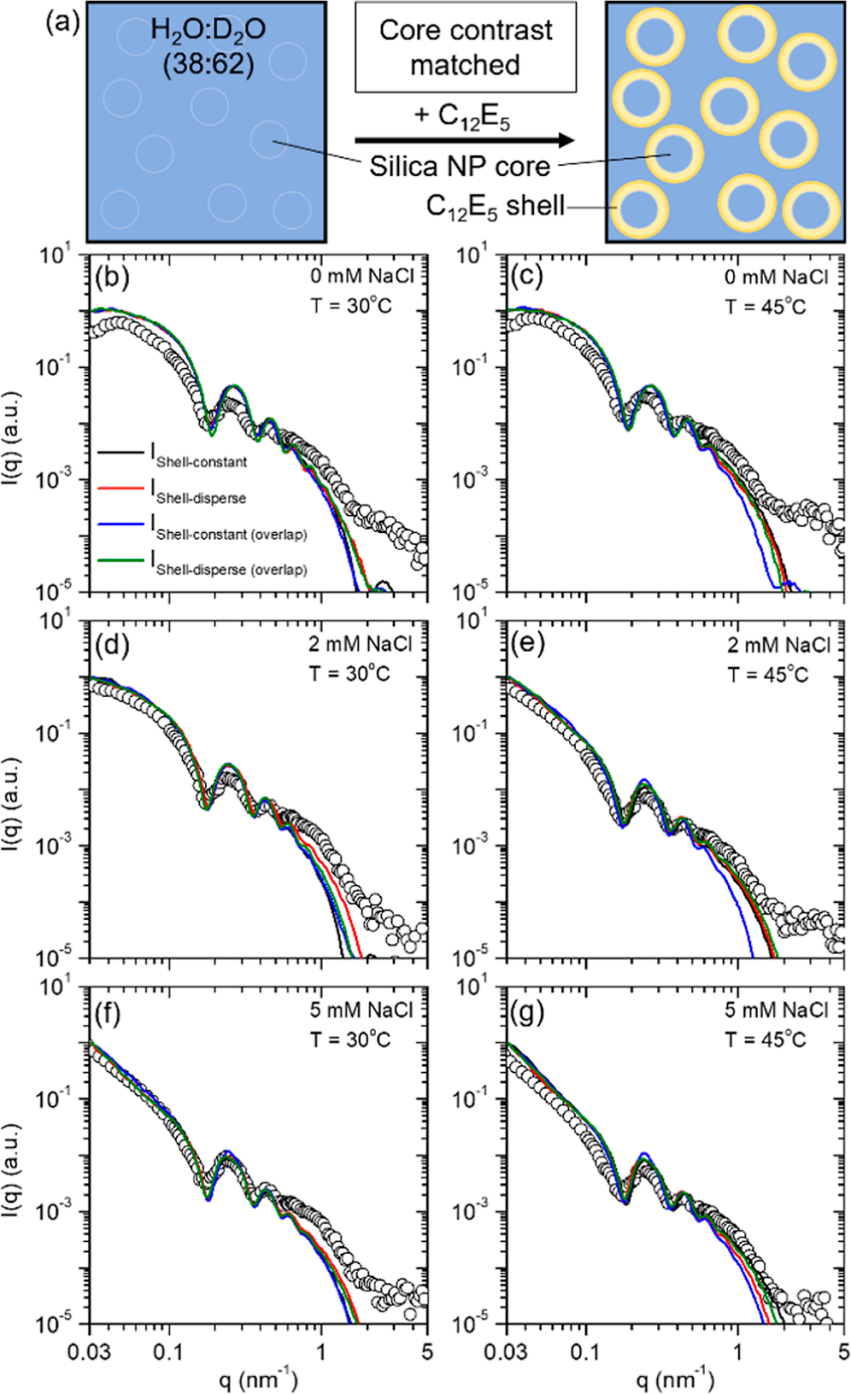
(a) Schematic representation of the surfactant adsorption and shell formation on silica NPs. Here, the SLD of the H_2_O/D_2_O solvent matches the SLD of the silica NPs such that the neutrons scatter only from the surfactant shell. (b–g) Experimental SANS data (circles) with increasing temperature and salinity and corresponding best fit computed scattering using CREASE analysis to test four potential scenarios, namely, shell thickness is constant among all NPs (black), shell thickness exhibits dispersity (red), shell thickness is constant but can overlap with other shells (blue), and shell thickness exhibits dispersity and can overlap with other shells (green).

To probe the changes in the surfactant self-assemblies, the SLD of the silica NP (3.7 × 10^–4^ nm^–2^) core was selectively contrast-matched using the 38:62 H_2_O/D_2_O mixture as a solvent. In the absence of added C_12_E_5_, a *q*-independent scattering is observed from the silica NP dispersion, highlighting contrast matching of the NPs using the H_2_O/D_2_O mixture (Figure S4). Upon the addition of C_12_E_5_ in the contrast-matched silica NPs, the primary oscillations at *q* ∼ 0.2 nm^–1^ emerge, highlighting the presence of the C_12_E_5_ surfactant on the surface of silica NPs (Figure 4b–g). The SANS was performed on 1 wt % silica NP dispersion containing a fixed amount of C_12_E_5_ such that Γ = 4.5 μmol m^–2^, which is equivalent to 0.9Γ_*m*_ (at 20 °C). Hence, nearly all surfactant molecules were present on the surface of silica NPs, and no significant amount of free surfactant existed in the solvent. The SANS profiles obtained for the silica–C_12_E_5_ mixture under silica contrast matching are shown in black circles in Figure 4b–g for 0, 2, and 5 mM NaCl at 30 °C (<*T*_c_) and 45 °C (>*T*_c_). Regardless of the temperature and concentration of the NaCl, all tested dispersions showed an oscillation at *q* ∼ 0.2 nm^–1^, highlighting the adsorption of surfactant molecules on the NPs. We find a significant change in the low-*q* scattering upon simultaneously increasing the dispersion salinity and temperature, which is indicative of the aggregation of the silica NPs. Note that the absolute scaling of the SANS data was not feasible in our experiments due to the aggregation of silica NPs and corresponding settling, which resulted in a decrease in the concentration of the NPs over time in scattering volume. The total scattering intensity from the silica–C_12_E_5_ mixture under silica contrast-matched conditions is given as^52^
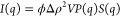
2where ϕ is the volume fraction of the scattering object, here surfactant, Δρ is the SLD contrast between the surfactant and the H_2_O/D_2_O mixture, *V* is the volume of the scattering object, and *P*(*q*) and *S*(*q*), respectively, are the form factor and structure factor of the self-assemblies formed by the surfactant molecules. The *P*(*q*) and *S*(*q*) could be changed with increasing temperature and salinity, as the adsorbed surfactant may change its configuration on the surface of the NPs. As a result, the conventional form factor model that works for the dispersed state may not be applicable in the aggregated state, making such a conventional analytical model fit-based analysis of the SANS profile challenging. Therefore, we use a machine learning (ML)-based computational reverse-engineering analysis for scattering experiments (CREASE) approach recently developed by the Jayaraman group to analyze the SANS data.^53−62^ In particular, the recent work with “*P*(*q*) and *S*(*q*) CREASE”^59^ is used for the analysis of the scattering profiles in this study. CREASE-based analysis produces the best fit computed scattering profile(s) while working with four different hypotheses about the adsorbed surfactant “shell” layer on the NPs with varying salinity and temperature (Figure 4b–g), namely, (i) shell thickness is the same across all NPs (black)—*I*_Shell-constant_, (ii) shell thickness across various NPs exhibits dispersity (red)—*I*_Shell-disperse_, (iii) shell thickness is the same across all NPs with potential overlap among the shells (blue)—*I*_Shell-constant (overlap)_, and (iv) shell thickness on various NPs exhibits dispersity with potential overlap among the shells (green)—*I*_Shell-disperse (overlap)_. The choice of the four potential scenarios is based on the previous reports, where C_*n*_E_*m*_ surfactants were shown to form a shell structure on silica NPs,^1,25,39^ and the overlapping of the shell potentially could occur during the aggregation of NPs.^63^

At 0 mM NaCl, the SANS data show a pronounced peak at low-*q* (*q* < 0.7 nm^–1^), indicating the ordering of the silica–C_12_E_5_ particles due to long-range repulsions that does not match with the CREASE-computed scattering profile because the CREASE platform, as of now, is designed for analyzing small-angle scattering from amorphous (positionally disordered) materials. As salinity increases, the silica–C_12_E_5_ particles become more disordered, enabling closer matches to the CREASE computed scattering profile at low-*q* values. To determine which of the four scenarios’ best fit computed scattering profiles from CREASE analysis is the closest match to the SANS data, we calculate the χ^2^ error.^54,59^ The computed scattering profile from CREASE analysis that treats the surfactant shell as varying in thickness among the NPs (case ii) achieves the closest match to the experimental SANS data for the 0 and 2 mM NaCl conditions for both solution temperatures considered. At the 5 mM NaCl condition, the computed scattering profile from CREASE analysis that treats the surfactant shell as varying in thickness among the NPs and allows overlap between neighboring surfactant shells (case iv) possesses the closest match to the experimental SANS data at both temperatures. This scattering analysis agrees with the transmittance results in Figure 3 that demonstrated minimal to low aggregation at 0 and 2 mM NaCl and substantial aggregation at 5 mM NaCl for the temperatures considered. Furthermore, the CREASE analysis suggests that at 5 mM NaCl, the surfactant shell undergoes a structural change, allowing nearby, aggregating shells to overlap or merge.

### 3.6 Interaction between Silica NPs: SANS with Shell Contrast-Matched

**Figure 5:**
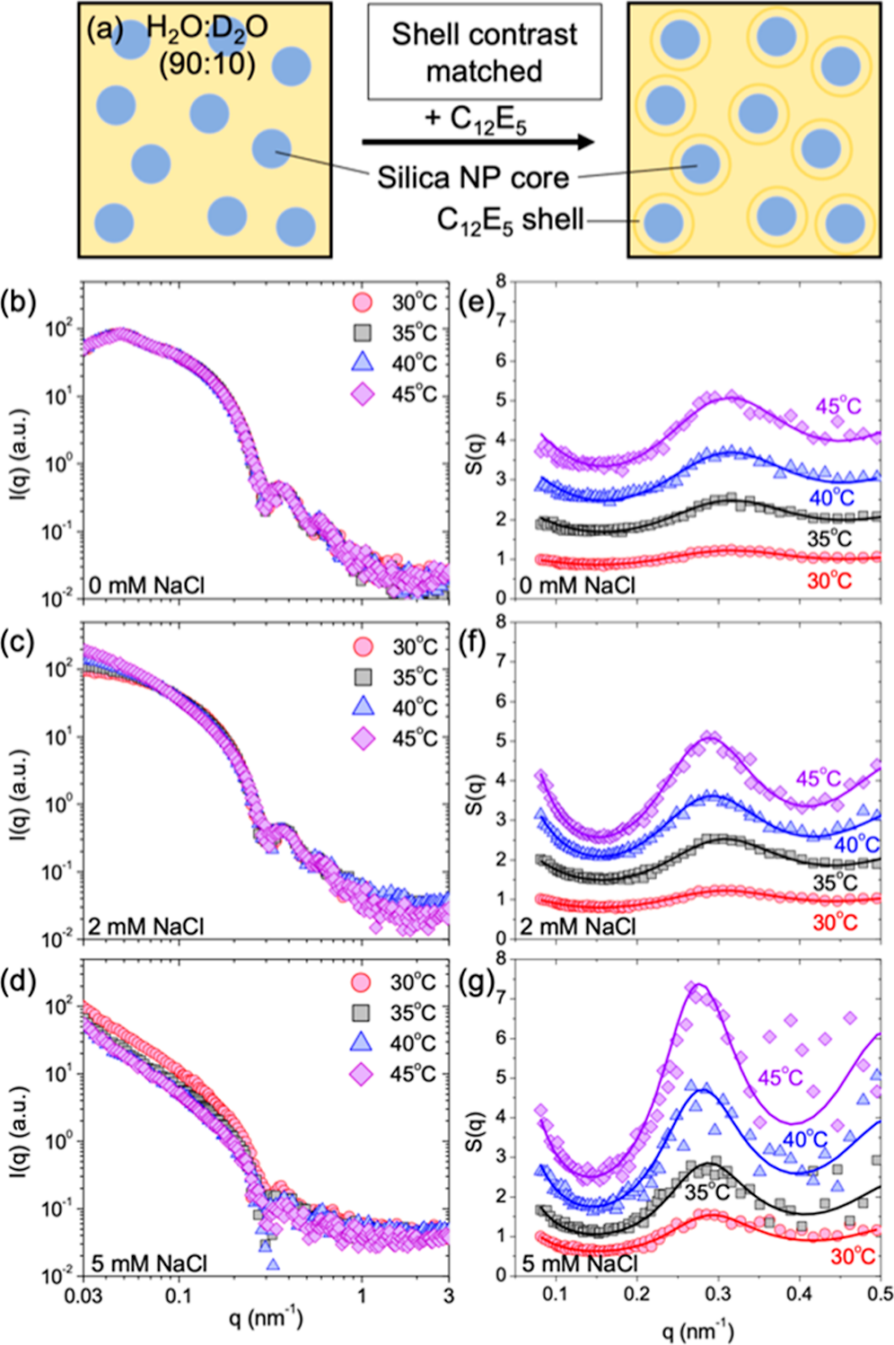
(a) Schematic of the C_12_E_5_-coated silica NP dispersion in the H_2_O/D_2_O mixture matching the SLD of the surfactant shell. (b–d) SANS profiles for the silica–C_12_E_5_ mixture under surfactant contrast-matched conditions in the presence of (b) 0, (c) 2, and (d) 5 mM NaCl at 30, 35, 40, and 45 °C. The distinction in scattering intensity at *q* < 0.07 nm^–1^ at different temperatures in (c) and (d) can be attributed to the aggregation of silica NPs and corresponding contribution of the structure factor to the overall scattering profile. (e–g) Structure factor, *S*(*q*) profiles (discrete points), and corresponding fits using the SWPY model (solid lines) for C_12_E_5_-coated silica NPs in the presence of (e) 0, (f) 2, and (g) 5 mM NaCl at 30, 35, 40, and 45 °C. The *S*(*q*) is obtained from the scattering profiles shown in (b–d) using eq 3. The individual *S*(*q*) curves are shifted vertically for clarity and better visualization.

The change in temperature and addition of NaCl did not prevent surfactant adsorption or initiate the desorption of C_12_E_5_ from the surface of silica NPs. However, the increase in temperature and dispersion salinity alters the interparticle interactions between surfactant-coated silica NPs. We identify the change in interparticle interactions by performing SANS experiments in the 90:10 H_2_O/D_2_O mixture matching the SLD of the hydrated surfactant shell (1.43 × 10^–5^ nm^–2^). Under these SLD contrast conditions, the neutrons scatter solely from the silica core (Figure 5a). The concentration of the surfactant was fixed at Γ = 4.5 μmol m^–2^, and the amount of NaCl in the dispersion was 0, 2, and 5 mM. The SANS profiles were measured at 30, 35, 40, and 45 °C. Note that the C_12_E_5_-coated silica NPs at 5 mM NaCl are strongly aggregated (discussed below), which settle within the sample cell, leading to a decrease in concentration of the NPs in the neutron beam over time. The impact of this decrease in effective concentration of the NP aspect can be observed in Figure 5d, where the SANS data show significant spread (noise) at *q* > 0.2 nm^–1^.

The SANS profiles for the silica–C_12_E_5_ mixture under surfactant contrast-matched conditions with increasing salt concentration at various temperatures are shown in Figure 5b–d. At 0 mM NaCl, the scattering profiles at all tested temperatures are similar to the scattering curve of bare silica in D_2_O (Figure 1a). This observation indicates that the silica NPs with the C_12_E_5_ shell remain colloidally stable with increasing temperature in the absence of salt. Upon the addition of 2 and 5 mM NaCl, a temperature-dependent change in the low-*q* slope (*q* < 0.07 nm^–1^) of scattering curves is observed. This change in scattering profiles in the low-*q* region follows the relation *I*(*q*) ∝ *q*^–*n*^ (Figure S5)^64^ where *n* is the dimensionality of the aggregates formed by the silica NPs.^65,66^ Generally, the value of *n* ∼ 0 indicates non-aggregated spheres, *n* ≤ 3 suggests the presence of mass-fractal aggregates, and *n* ∼ 4 is the Porod scattering from large aggregates/structures. The values of the parameter *n* obtained from the SANS profiles at 2 and 5 mM NaCl with increasing temperatures (Figure S5) are given in Table 2.

**Table 2:** Low-*q* Slopes, i.e., Values of Parameter *n,* for C_12_E_5_-Coated Silica NP in H_2_O/D_2_O Matching the SLD of the Surfactant upon Increasing NaCl Concentration and Temperature

*T* (°C)	*n* at 2 mM NaCl	*n* at 5 mM NaCl
30	0.1	1.9
35	0.2	2.2
40	0.6	2.6
45	0.9	2.8

The value of *n* increases with increasing NaCl concentration and temperature (Table 2), indicating an increase in the dimensionality of the fractal aggregates formed by silica NPs. The absolute value of *n* for dispersions with 5 mM NaCl is higher than that in 2 mM NaCl in all tested temperatures, indicating the formation of silica aggregates with larger fractal dimensions. The aggregation behavior of C_12_E_5_-coated silica NPs and the corresponding change in interparticle interactions upon increasing NaCl concentration and temperature are quantitively studied by extracting the structure factor from the scattering profiles shown in Figure 5b–d. The structure factor, *S*(*q*), for the dispersion of C_12_E_5_-coated silica NPs is obtained as^65,67^
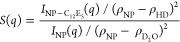
3where  is the total scattering intensity from C_12_E_5_-coated silica NPs in 90:10 H_2_O/D_2_O and () is the SLD contrast between silica NPs and the solvent; and  is the scattering intensity from silica NP dispersion in D_2_O (Figure 1a) with () as the SLD contrast.

The attraction between silica NPs and the corresponding degree of aggregation increases with increasing concentration of NaCl and temperature. However, the form factor of the silica NPs does not change with increasing salinity and temperature, allowing us to use the conventional analytical model fit approach to interpret the *S*(*q*). The corresponding *S*(*q*) profiles of silica NPs in surfactant contrast-matched conditions with increasing salinity and temperature are shown in Figure 5e–g (discrete points). The *S*(*q*) shows an oscillation at *q* ∼ 0.3 nm^–1^, whose amplitude increases with increasing temperature and increasing concentration of added NaCl (Figure 5e–f). The presence of oscillation is a characteristic of the existence of an immediate neighbor due to short-range attractive interactions between silica NPs. The enhancement in the amplitude of the oscillation at *q* ∼ 0.3 nm^–1^ highlights the increase in the number of nearest neighbors owing to the stronger attraction between the particles with increasing concentration of NaCl and temperature. The net interaction potential between silica NPs within the aggregates can be obtained by fitting the *S*(*q*) to a square-well potential model, which describes the short-range attractive interactions between the hard spheres. This square-well pair potential is given as^67,68^
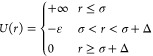
4where  is the diameter of silica NPs and  and  are the well-depth and well-width, respectively. The square-well potential can be calculated from the Ornstein–Zernike equation with Percus-Yevick approximation, and this square-well Percus-Yevick (SWPY) model is simplified to a sticky-hard-sphere (SHS) model when  0 and (69) The *S*(*q*) in the SHS model is represented as a function of volume fraction of particles within the aggregates (*f*_p_) and a stickiness parameter  which is given as  where  is the Boltzmann constant and *T* is the temperature. The dimensionless parameter  is linearly related to the temperature, and the  depicts the stickiness of the particles where  represents non-sticky hard spheres. The SHS model predicts the phase separation in sticky hard spheres at  > 10.2,^68^ which in our case translates to aggregation. Further information on this analytical model is provided in previous publications.^65,70^

**Figure 6:**
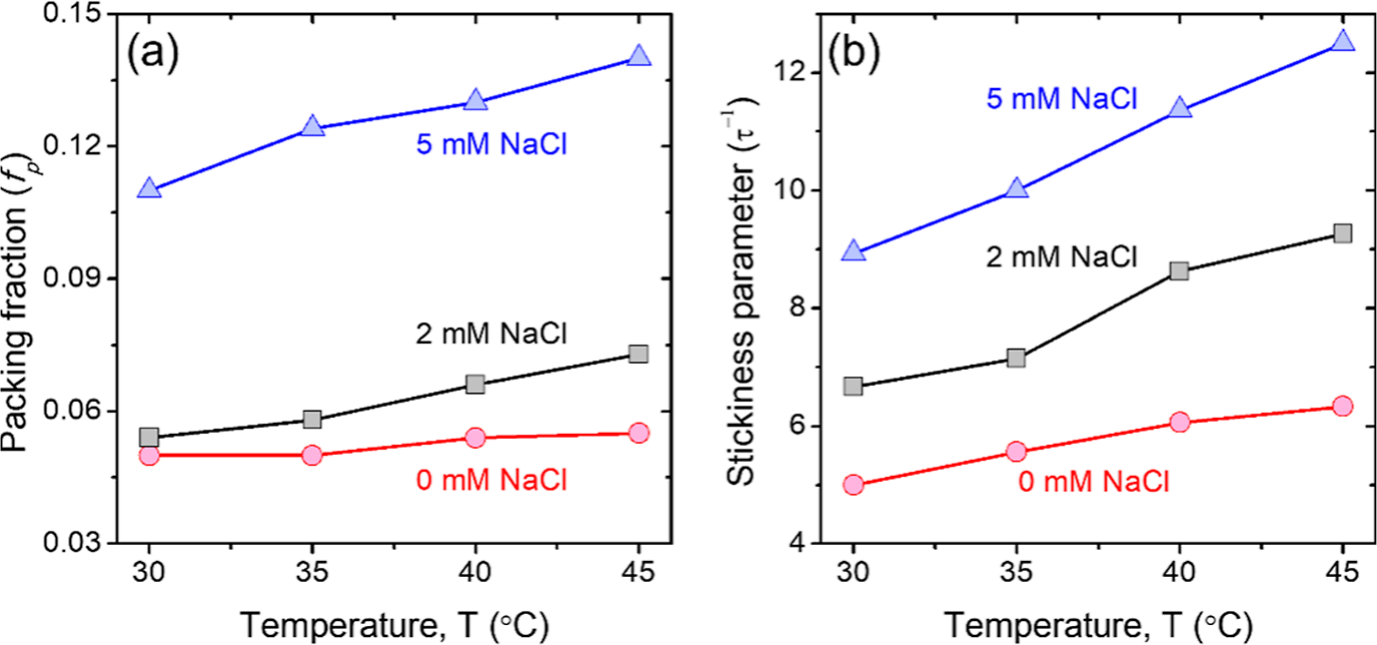
SWPY fit parameters obtained by analysis of the structure factor data for silica–C_12_E_5_ complexes, as shown in Figure 5. The fit parameters are (a) packing fraction of silica NPs within aggregates and (b) stickiness of the NPs are plotted as a function of temperature at increasing concentrations of NaCl.

The S(*q*) data for increasing concentration of NaCl and increasing temperature are fitted using the SWPY model, as shown in Figure 5e–g (solid lines). The theoretical model effectively represents the experimental results of the *S*(*q*). The fitting parameters *f*_p_ and  are plotted as a function of temperature for 0, 2, and 5 mM of NaCl in Figure 6. We find that both *f*_p_ and  increase with temperature at a given salt concentration, highlighting the formation and densification of the aggregate structure, which agrees with the transmittance measurements shown in Figure 3. For all tested NaCl concentrations at *T* < *T*_c_,  < 10.2, i.e., C_12_E_5_-coated NPs, remained stable below the *T*_c_ of the surfactant. Furthermore,  remains below its critical value for all tested temperatures at 0 and 2 mM NaCl. This observation combined with the trends in the transmittance of the dispersions (Figure 3) highlights that silica NPs exist in a weakly aggregated state for 0 and 2 mM at *T* > *T*_c_. The stronger aggregation of silica NPs is observed in dispersion with 5 mM NaCl at *T* > *T*_c_, where the *f*_p_ is the highest (Figure 6a). The presence of aggregates in 5 mM NaCl is also corroborated by transmittance measurements that decrease to <40% at *T* > *T*_c_ (Figure 3).

**Figure 7:**
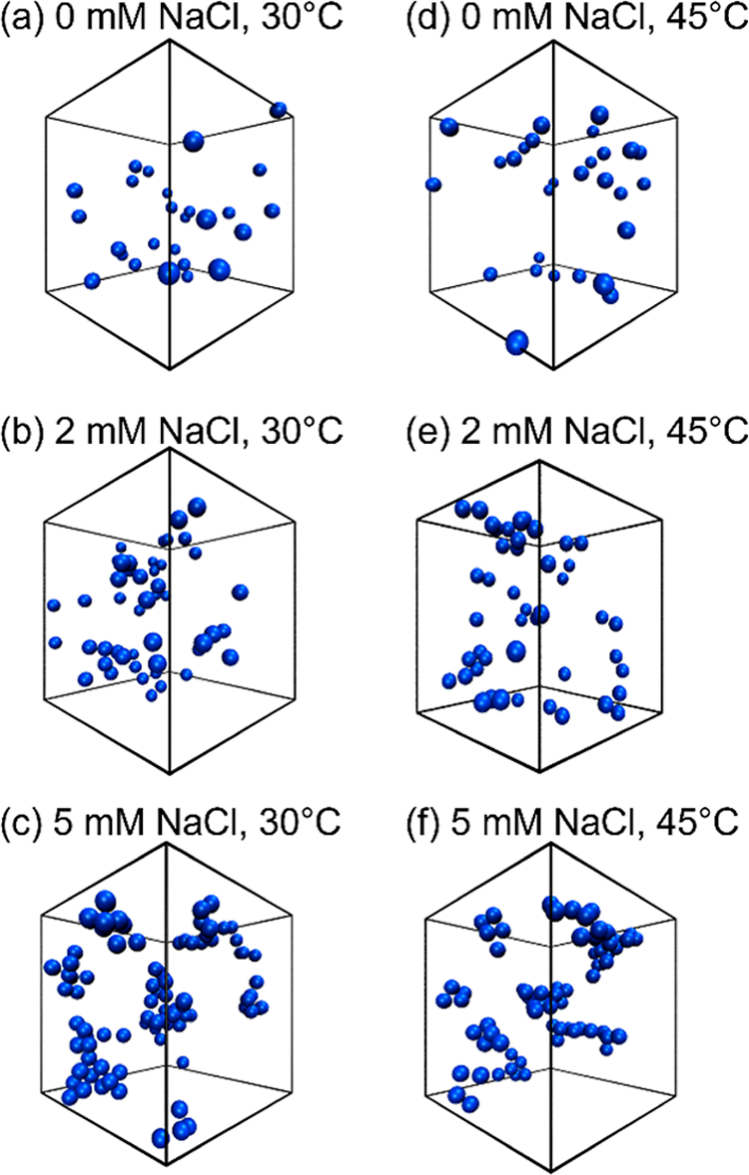
Representative snapshots of the real space 3D structure of C_12_E_5_-coated silica NPs (blue spheres) at various NaCl concentrations at (a–c) 30 °C and (d–f) 45 °C. These 3D structures are obtained as one of the outputs of the CREASE analysis of the experimental SANS data performed in the H_2_O/D_2_O mixture matching the SLD of the silica NPs. The images show the densification of the aggregates in 5 mM NaCl.

The observed changes in  and *f*_p_ with added NaCl and increasing temperature highlight the synergy between the two experimental parameters in driving the aggregation of C_12_E_5_-coated silica NPs. The aggregation behavior can be attributed to the hydrophobic interactions between C_12_E_5_-coated NPs in the presence of NaCl, where the water molecules bound to the headgroup of the surfactant molecules are released upon increasing the temperature *T* > *T*_c_. Additionally, the dissociation of NaCl in water establishes an ionized solvent field and decreases the solubility of alkyl tails of the surfactant molecules, hindering the solvation of the surfactant with water and dehydrating the C_12_E_5_ on the silica surface.^71−73^ Based on the SANS measurements under shell contrast-matched conditions, we can conclude that weak aggregation of the surfactant-coated silica NPs occurs in 2 mM NaCl at *T* > *T*_c_, whereas much denser aggregates are formed in 5 mM NaCl. We also observe such densification of the aggregates in the analysis of the SANS data under silica contrast-matched conditions (i.e., scattering from surfactant shells) using CREASE. The snapshots obtained from the CREASE analysis of SANS data of dispersions containing 0, 2, and 5 mM of NaCl at 30 and 45 °C are shown in Figure 7. The images are obtained from the CREASE analysis of SANS using the form factor of the surfactant shell of constant thickness overlapping with the shell(s) of neighboring particles. The reconstructed representative real space images demonstrate a clear synergy between salinity and temperature in facilitating the aggregation of C_12_E_5_-coated silica NPs.

### 3.7 Property of Dispersions: Change in Viscosity with Salinity and Temperature

**Figure 8:**
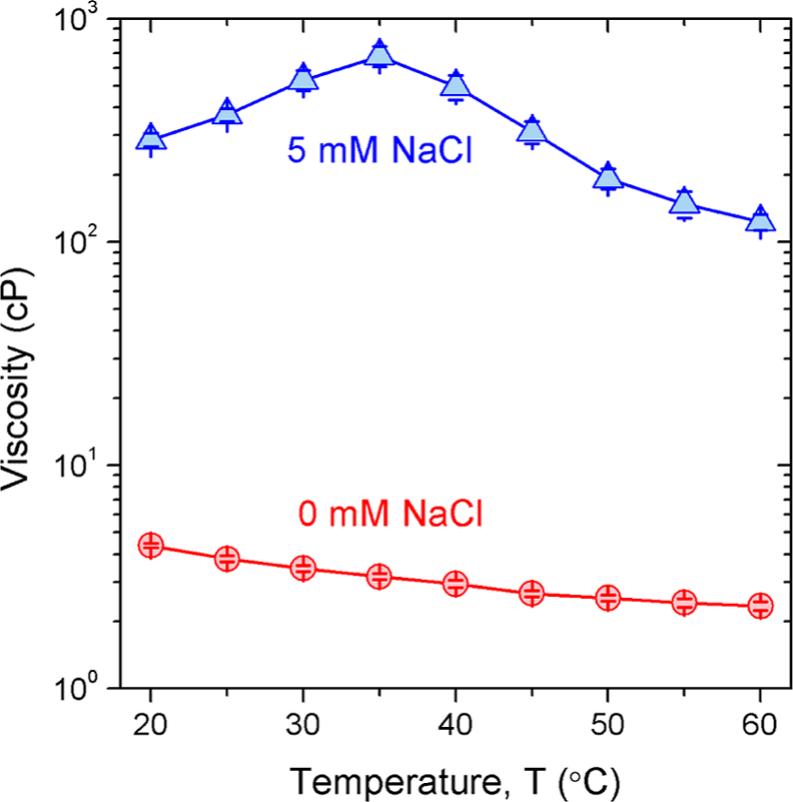
Viscosity of C_12_E_5_-coated silica NPs (10 wt %) as a function of temperature in 0 mM (triangles) and 5 mM NaCl (circles). The measurements were performed at a constant shear rate of 2.64 s^–1^. The dispersion becomes significantly more viscous upon the addition of NaCl and shows a non-monotonic change with temperature due to the densification and settling of aggregates upon increasing temperature.

Temperature and the addition of salt are key factors governing the viscosity of any solution. Generally, viscosity decreases with increasing temperature and increasing NaCl concentration in water.^74^ However, due to the observed NP aggregation, we find an increase in viscosity upon the addition of 5 mM NaCl to the C_12_E_5_-coated silica NP dispersion. The viscosity of the bare silica NPs and C_12_E_5_-coated silica NP dispersions was measured using a DV2T viscometer (Brookfield) at various temperatures. A typical viscosity measurement was performed using the aqueous solution containing 10 wt % silica NPs and C_12_E_5_ surfactant equivalent to Γ = 0.9Γ_*m*_ (at 20 °C) in the presence of NaCl such that no unadsorbed surfactant was present in the solvent. The shear rate during the measurement was kept constant at 2.64 s^–1^, and the temperature was increased from 20 to 60 °C. The viscosity measured at various temperatures at 0 and 5 mM NaCl concentration is shown in Figure 8. The viscosity of the water containing C_12_E_5_-coated silica NPs and 0 mM NaCl decreases with increasing temperature. As discussed in Section 3.4, at 0 mM NaCl, the dispersion is in a single phase where the increase in temperature leads to a decrease in viscosity due to weakening of the attractions between the layers of water molecules. However, the viscosity of the C_12_E_5_-coated silica NPs containing 5 mM NaCl is two orders of magnitude higher at all temperatures than in the absence of NaCl. Note that the shear rate during the viscosity measurements was kept constant; therefore, the observed changes in viscosity with salinity and temperature are primarily the result of the change in the self-assembled state of the C_12_E_5_-coated silica NPs in the aqueous medium.

We find a non-monotonic change in the viscosity with temperature for 5 mM NaCl dispersion, where it first increases upon increasing temperature from 20 to 35 °C and then decreases from 35 to 60 °C. The increase in viscosity upon increasing temperature up to 35 °C is the result of the enhancement in the aggregation of the NPs. The decrease in viscosity observed at temperatures above 35 °C may be attributed to multiple factors, including the settling of aggregates, reconfiguration of the aggregate structure driven by temperature changes, and ordering induced by shear in the dispersion. However, based on our current experiments, we cannot definitively determine the cause of the non-monotonic change in viscosity with temperature. To better understand this phenomenon, further rheology and SANS experiments will be necessary. We note that such viscosity changes are not observed in bare silica NP dispersions containing 0 and 5 mM NaCl (Figure S6), highlighting the critical role of adsorbed C_12_E_5_ molecules in programming viscosity. These experiments demonstrate the synergistic role of dispersion salinity and temperature in promoting aggregation of C_12_E_5_-coated silica NPs correspondingly impacting the properties, here viscosity of the aqueous dispersion.

## 4 Conclusions

This study identified the synergistic effects of salinity and temperature on enhancing the adsorption of C_12_E_5_ molecules on silica NPs and promoting the aggregation of the NPs. We showed that the maximum amount of C_12_E_5_ that can be adsorbed on silica NPs increases with increasing temperature and the amount of added NaCl. This increase in maximum surface excess is driven by the dehydration of the C_12_E_5_ headgroup and increase in effective hydrophobicity of the surfactant tail. The silica NPs with adsorbed C_12_E_5_ show rich salinity and temperature-dependent phase behavior. Increasing salinity and temperature reduces the transmittance of the mixture, highlighting the formation of large aggregates. We uncover the structures formed by the surfactant and silica aggregates by performing SANS experiments under silica and surfactant shell contrast-matched conditions. Based on our analysis using CREASE and the sticky hard sphere model, we demonstrate the densification of the aggregates of dispersions containing 5 mM NaCl at temperatures above the *T*_c_ of C_12_E_5_. We attribute the observed aggregation and subsequent densification of the aggregates to the enhanced hydrophobic attraction between the C_12_E_5_-coated silica NPs. We further uncover the structure–property relationship by correlating the change in the aggregation state of C_12_E_5_-coated silica NPs with the viscosity of the aqueous solution. This study highlights the impact of solvent parameters, here salinity and temperature, on the adsorption of ethoxylated surfactants on hydrophilic NPs and lays a foundation to begin to engineer colloidal dispersion with programable viscosity to be used in inks and paints.

## Supporting Information Available

Surface tension calibration curves of C_12_E_5_ in bulk solution for adsorption isotherms, *T*_c_ of C_12_E_5_ at various NaCl concentrations, stability of bare silica NPs in the presence of NaCl, contrast matching of silica NPs using the H_2_O/D_2_O mixture as a solvent, slope  of the low- region determined by analysis of SANS profiles in surfactant contrast-matched conditions, and viscosity of silica NPs in the presence and in the absence of NaCl (PDF)
