# Supplementary material for: Synergistic Role of Temperature and Salinity in Aggregation of Nonionic Surfactant-Coated Silica Nanoparticles
Source: Langmuir. 2023 Apr 13;39(16):5917–28. doi: 10.1021/acs.langmuir.3c00432 (PMC10134496; doi:10.1021/acs.langmuir.3c00432)
Supplement: Supplementary file 1 — la3c00432_si_001.pdf [file la3c00432_si_001.pdf]

Supporting Information

Synergistic role of temperature and salinity in aggregation of nonionic surfactant coated silica nanoparticles

Yingzhen Ma[1], Christian Heil[2], Gergely Nagy[3], William T. Heller[3], Yaxin An[1], Arthi Jayaraman[2], Bhuvnesh Bharti[1,*]

[1]Cain Department of Chemical Engineering, Louisiana State University, Baton Rouge, Louisiana 70803, USA
[2]Department of Chemical and Biomolecular Engineering, University of Delaware, Newark, Delaware 19716, USA
[3]Neutron Scattering Division, Oak Ridge National Laboratory, Oak Ridge, Tennessee 37831, USA

*Corresponding author's email: bbharti@lsu.edu

Content	Page
Surface tension calibration curves of $C_{12}E_5$ in bulk solution for adsorption isotherms	S2
Cloud point of $C_{12}E_5$ at increasing NaCl concentration	S3
Stability of bare silica NPs in the presence of NaCl	S4
SANS contrast matching of silica NPs using H_2O:D_2O mixture as solvent	S5
Slope (n) of the low-q region determined by analysis of SANS profiles in surfactant contrast matched condition	S6
Viscosity of silica NPs in the presence and in the absence of NaCl	S7

I. Calibration curve of $C_{12}E_5$ in bulk solution for adsorption isotherms

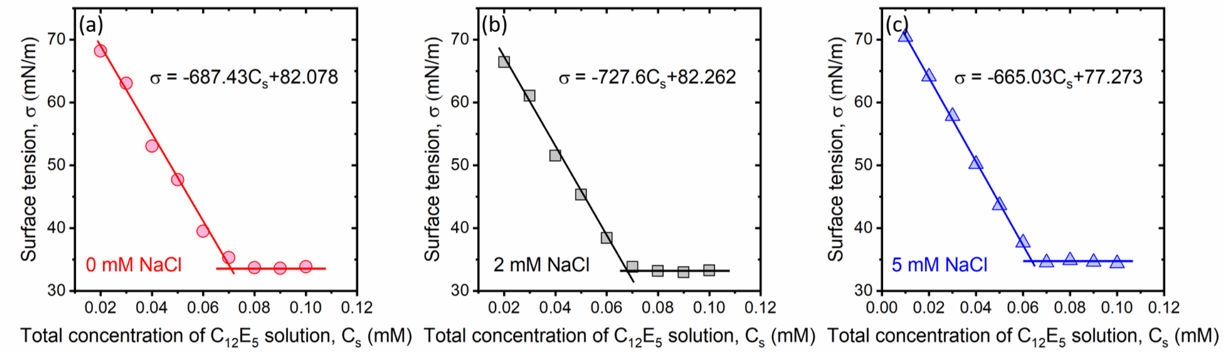

Figure S1. Surface tension calibration curve of $C_{12}E_5$ solution in the presence of (a) 0 mM, (b) 2 mM, and (c) 5 mM NaCl. The discrete points are the experimentally measured surface tension values of a given surfactant solution, and the lines are the linear fits to the experimental data. The equation of line representing the surface tension decrease in the range $c_s < cmc$ is given in the plot, where σ and C_s are the surface tension and concentration of the supernatant, respectively.

II. Change in cloud point of $C_{12}E_5$ with increasing salinity

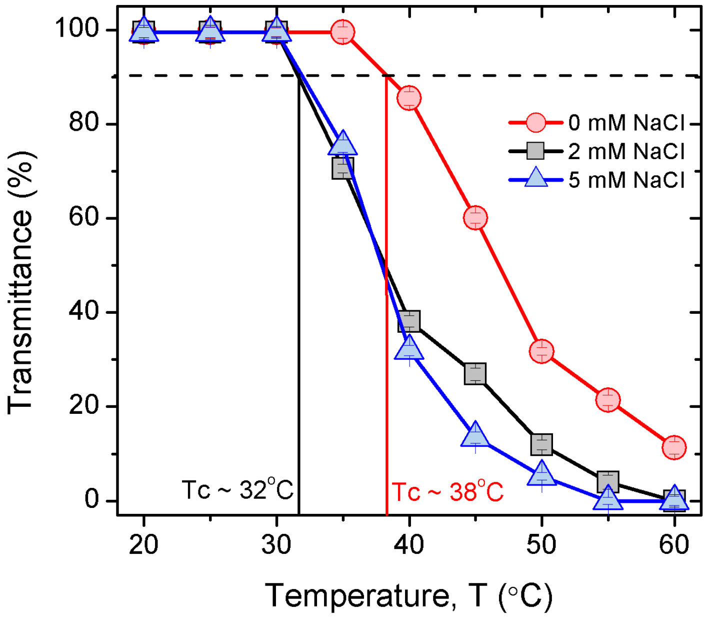

Figure S2. The change in transmittance of 2.6 mM $C_{12}E_5$ solution in the absence of silica NPs at 0 mM, 2 mM, and 5 mM NaCl. The cloud point of the surfactant decreases from 38 °C to 32 °C upon the addition of NaCl. The T_c values are estimated by assuming 90% transmittance as the onset of cloud point.

III. Stability of bare silica NPs

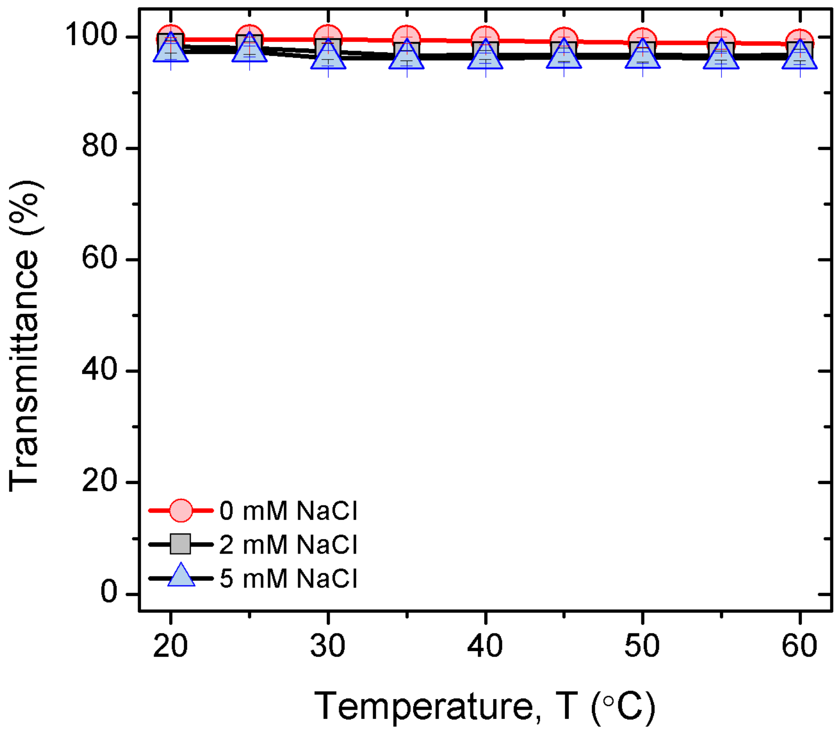

Figure S3. Transmittance of silica NPs in the absence of $C_{12}E_5$ but in the presence of 0 mM, 2 mM, and 5 mM NaCl. The lack of change in transmittance upon increase in salinity and temperature points to the stability of the bare silica NPs in aqueous dispersion.

IV. Contrast matching of silica NPs using H_2O:D_2O mixture as solvent

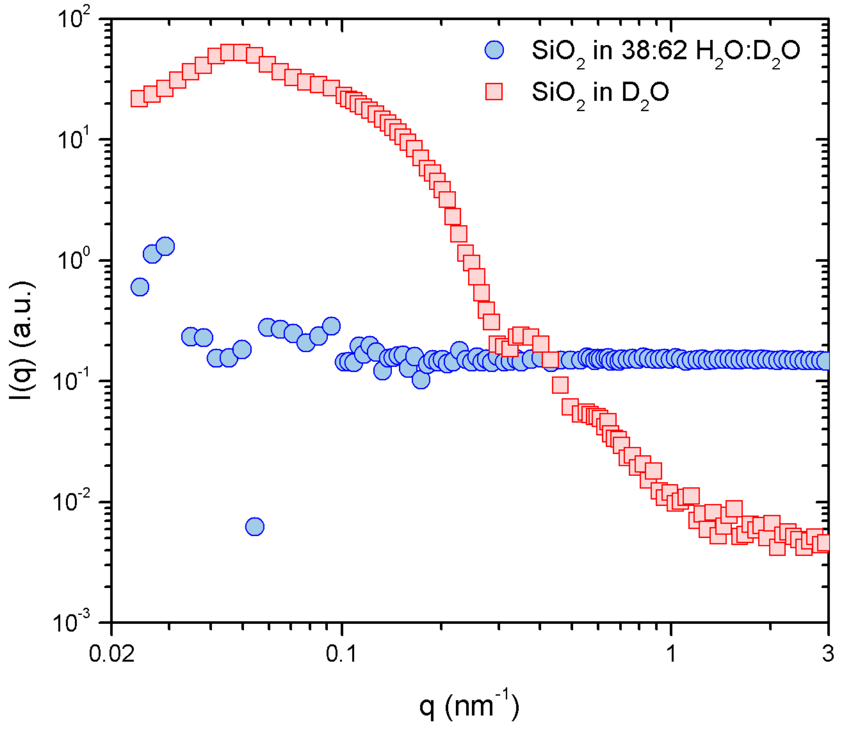

Figure S4. SANS profile of 1 wt% silica NPs in D_2O and in H_2O:D_2O mixture matching the scattering length density of the silica. The water vector independent scattering from NPs dispersed in H_2O:D_2O highlights completely contrast matching of the silica.

V. Slope n of the low-q region of silica NPs under $C_{12}E_5$ contrast matched condition

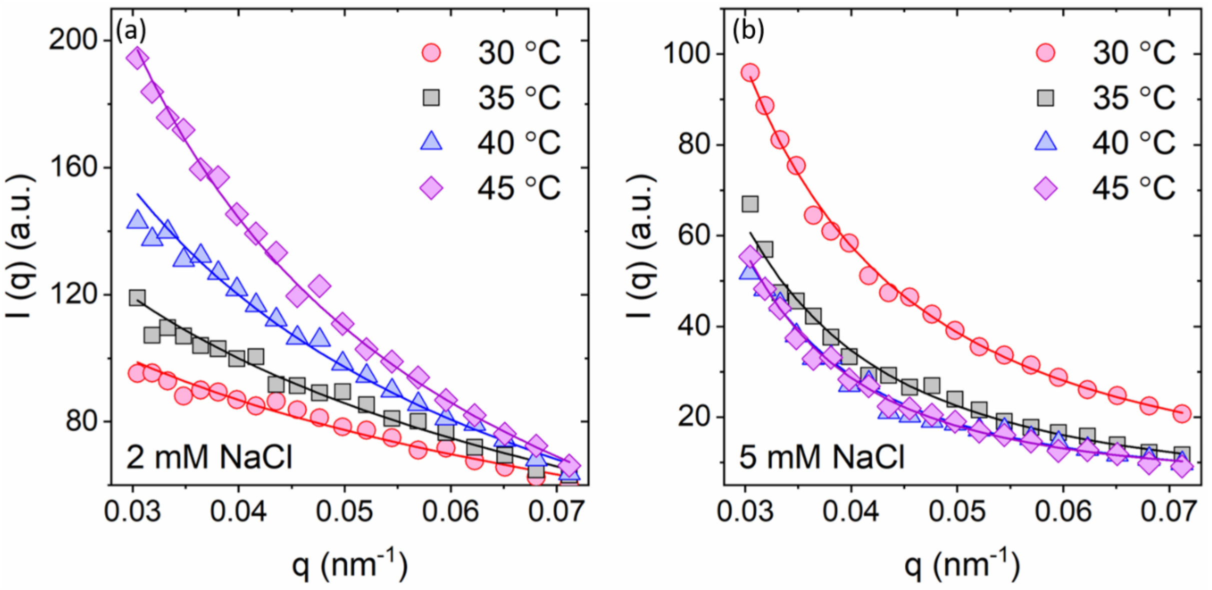

Figure S5. Experimental SANS profiles for $C_{12}E_5$ adsorbed silica NPs in presence of (a) 2 mM and (b) 5 mM NaCl at different temperature under surfactant contrast matched conditions. The lines represent fits to the low-q SANS profiles by $I(q) \propto q^{-n}$. The values of n are summarized in Table 2.

VI. Viscosity of silica NPs with different addition of salt

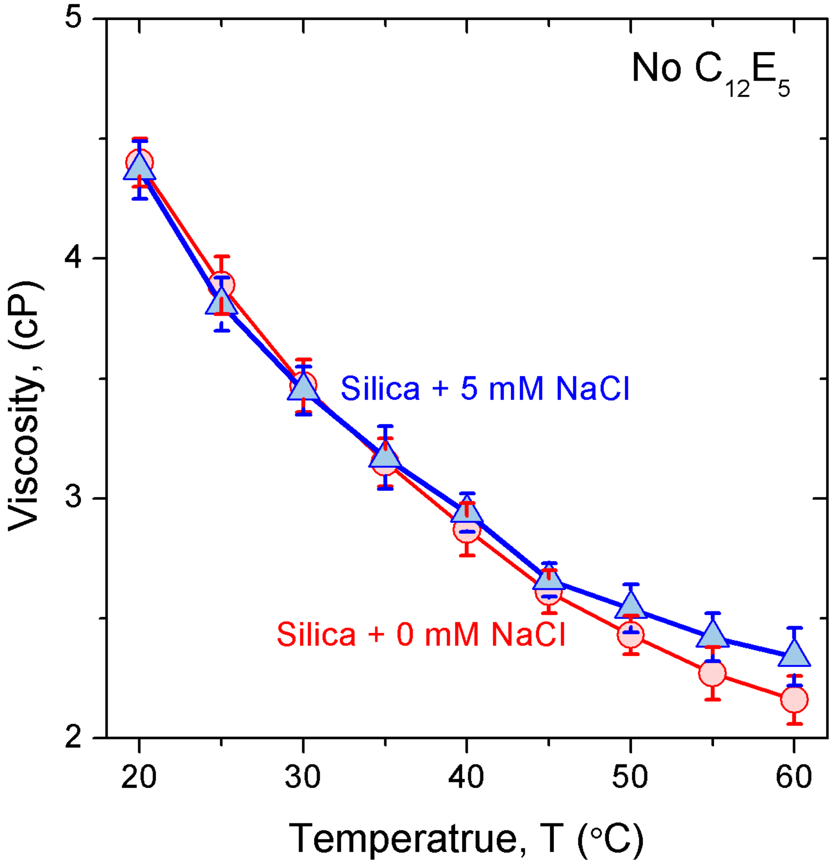

Figure S6. Viscosity of silica NPs in presence and in absence of NaCl upon increasing temperature from 20 °C to 60 °C.
